# FDG-PET parameters predicting mediastinal malignancy in lung cancer

**DOI:** 10.1186/s12890-016-0338-6

**Published:** 2016-12-08

**Authors:** M. Serra Fortuny, M. Gallego, Ll Berna, C. Montón, L. Vigil, M. J. Masdeu, A. Fernández-Villar, M. I. Botana, R. Cordovilla, R. García-Luján, E. Cases, E. Monsó

**Affiliations:** 1 Hospital Universitari Parc Taulí, Sabadell, Spain; 2 Departament de Medicina, Universitat Autònoma de Barcelona, Bellaterra, Spain; 3 Ciber de Enfermedades Respiratorias - Ciberes, Madrid, Spain; 4 Health Services Research on Chronic Diseases Network- REDISSEC, Madrid, Spain; 5 Complexo Hospitalario Universitario de Vigo, Vigo, Spain; 6 Complejo Asistencial de Salamanca, Salamanca, Spain; 7 Hospital Universitario 12 de Octubre, Madrid, Spain; 8 Hospital Universitari La Fe, Valencia, Spain

**Keywords:** FDG-PET, F-18 fluorodeoxyglucose positron emission tomography, Lung cancer, NSCLC, EBUS-NA, Endobronchial ultrasonography with needle aspiration

## Abstract

**Background:**

Staging of mediastinal lymph nodes in non-small cell lung cancer (NSCLC) is mandatory. The maximum Standard Uptake Value (SUVmax) obtained using F-18 fluorodeoxyglucose positron emission tomography (FDG-PET) is the best non-invasive technique available for this evaluation, but its performance varies from center to center. The aim of the present study was to identify FDG-PET predictors of mediastinal malignancy that are able to minimize intercenter variability and improve the selection of subsequent staging procedures.

**Method:**

A multicenter study of NSCLC patients staged through FDG-PET and endobronchial ultrasonography with needle aspiration (EBUS-NA) was performed using therapeutic surgery with systematic nodal dissection as gold standard. Intercenter variability and predictive power for mediastinal malignancy of different FDG-PET measures were assessed, as well as the role of these measures for selecting additional staging procedures.

**Results:**

One hundred and twenty-one NSCLC patients, of whom 94 (72%) had ≥1 hypermetabolic spots in the mediastinum, were included in the study. Mean SUVmax of the primary tumor was 12.3 (SD 6.3), and median SUVmax of the highest hypermetabolic spots in the mediastinum was 3.9 (IQR 2.4-7). Variability of FDG-PET measures between hospitals was statistically significant (*p* = 0.016 and *p* < 0.001 respectively), but lost significance when SUVmax in the mediastinum was expressed as a ratio or a subtraction from the primary tumor (SUVmax mediastinum/tumor, *p* = 0.083; and SUVmax mediastinum - tumor, *p* = 0.428 respectively). SUVmax mediastinum/tumor showed higher accuracy in the ROC analysis (AUC 0.77 CI 0.68-0.85, *p* < 0.001), and showed predictive power for mediastinal malignancy when using a 0.4 cutoff (OR 6.62, 95%CI 2.98-14.69). Sensitivities and negative predictive values of clinical staging through EBUS-NA attained values ranging between 57% and 92% after FDG-PET, which improved with additional techniques when the tumor had a diameter >3 cm and/or a SUVmax mediastinum/tumor ratio >0.4.

**Conclusion:**

The SUVmax mediastinum/tumor ratio is a good predictor of regional tumor extension in NSCLC. This measure is not influenced by intercenter variability and has an accuracy of over 70% for the identification of malignancy when using a 0.4 cutoff.

## Background

An accurate lymph node evaluation is mandatory in patients with lung cancer (LC) who do not present distant metastasis at their first examination. The implementation of F-18 fluorodeoxyglucose positron emission tomography (FDG-PET) has improved non-invasive LC staging, because this technique adds metabolic information to thoracic imaging and optimizes mediastinal sampling by improving targeting accuracy in comparison with computed tomography (CT) [1–7].

The maximum Standard Uptake Value (SUVmax) provides information on the metabolic activity of the primary tumor and its regional lymph nodes. It is usually increased in the mediastinum when lymph nodes show malignant infiltration, although high FDG uptake may also be observed in inflammatory diseases. Several studies have assessed the predictive power of SUVmax for identifying malignant extension of LC to lymph nodes in the mediastinum, and various cutoffs for mediastinal malignancy have been proposed, ranging from 2.5 to 5.3 [8–10]. The extensive use of these cutoffs, however, is limited by the variability in the SUVmax values recorded at different FDG-PET centers, which is reported to range between 10% and 15% [11, 12]. The use of ratios between SUVmax values in the mediastinum and the tumor has been proposed as a way of homogenizing the results obtained at different centers and allows the definition of cutoffs that may not be influenced by intercenter variability [13–15].

Although the sensitivity and specificity of imaging techniques for LC staging have improved with the introduction of FDG-PET, the technique lacks sufficient accuracy to guide treatment after the procedure, mainly because its sensitivity and negative predictive value (NPV) for malignancy have shown figures below 80% in most studies [6, 16–18]. Therefore, in accordance with current guidelines a biopsy is still needed to confirm lymph node involvement, except in peripheral lung cancers below 3 cm in diameter and negative results of imaging studies of the mediastinum in which case the probability of malignancy is very low [19–21]. Endoscopic techniques like endobronchial ultrasonography with needle aspiration (EBUS-NA) are used to confirm FDG-PET results. However, the NPV of this technique after FDG-PET has not been determined, and current guidelines recommend the performance of invasive techniques such as cervical mediastinoscopy after a negative EBUS-NA before therapeutic surgery, regardless of the FDG-PET imaging results.

The aim of the present study was to identify optimal predictors of mediastinal malignancy from a PET exam which are able to minimize the variability between hospitals and improve the selection of subsequent sampling procedures such as EBUS-NA. First, the variability and predictive power of absolute and relative SUVmax values obtained from the primary tumor and the mediastinum by FDG-PET were assessed in a multicenter LC study; then, the sensitivity and NPV of EBUS-NA were calculated according with FDG-PET results.

## Methods

### Design and patients

A prospective, multicenter study including patients with a diagnosis of non-small cell lung cancer (NSCLC) was conducted at five university hospitals between 2010 and 2012. Patients with suspected NSCLC were referred by their general practitioner to one of the participating hospitals for diagnosis and staging. Non-invasive staging examinations included a CT scan of the lung, mediastinum and upper abdomen using a multidetector-row spiral CT scanner, considering nodes in the mediastinum as abnormal when their short-axis diameter was greater than 10 mm, and a FDG-PET in patients with no suspected distant metastasis at first examination. Non-surgical pathological staging was performed by EBUS-NA, and patients with an EBUS-NA procedure negative for malignancy in the mediastinum were staged by esophageal ultrasound with needle aspiration (EUS-NA) and/or cervical mediastinoscopy when their thoracic imaging suggested mediastinal malignancy and/or their primary tumor was central or over 3 cm in diameter [19, 20]. All staging procedures were performed 1-3 weeks after the first hospital visit, to proceed to treatment not later that one month after that visit, according with fast-track protocols for lung cancer. Patients with hemorrhagic diseases or coagulation disorders were excluded from staging by EBUS-NA. Operability was assessed by physical examination, blood tests and lung function. Only patients with a diagnosis of NSCLC who were staged by CT, FDG-PET and EBUS-NA and treated surgically when staging results were negative were included in the study. Therapeutic surgery with systematic nodal dissection was performed after EBUS and mediastinoscopy negative for malignancy, and was considered the gold standard for the present study. The research protocol was approved by the reference regional ethics committee for the project (Institut de Recerca en Ciències de la Salut Germans Trias i Pujol, reference: FIS PS09/01612) and by the local ethics committees of all participating centers. Written informed consents were obtained from all participating patients.

### F-18 fluorodeoxyglucose positron emission tomography

FDG-PET with a integrated CT equipment (FDG-PET/CT) (4 hospitals) or performed just after CT (1 hospital) (Phillips, Amsterdam, The Netherlands; or General Electric, Fairfield, Connecticut, United States of America), was used to stage the mediastinum at each hospital in patients with no suspected distant metastasis at first examination. Prior to 18 F-FDG administration, patients fasted for at least six hours and plasma glucose level was confirmed to be inferior to 175 mg/dL. The patient received intravenously standard activity 3,7 MBq of 18 F-FDG per kg of weight and rested 60 minute for the scheduled uptake period. FDG-PET images were acquired from mid-skull to the mid-thigh with the patient supine, reconstructed with a standard iterative algorithm, and reformatted into axial, coronal, and sagital views. Administered activity, time of administration, and patient's body weight were recorded for calculation of maximum standardized uptake values (SUV max). Recovered images were interpreted by a nuclear medicine physician who evaluated both the appearance of the primary lung tumor and the hypermetabolic lymph nodes in the mediastinum. Standard uptake values of examined regions were assigned by the software of the equipment used in each hospital. To quantify the maximal metabolic activity of lymph nodes in the mediastinum their highest SUVmax was measured, as an absolute value, as a ratio between this value and the SUVmax of the primary tumor, and after the subtraction of this second value from this highest SUVmax in the mediastinum [13–15]. Homogeneity of FDG-PET between hospitals was assessed through comparisons of the SUVmax values of primary lung tumors and of lymph node regions with the highest SUVmax in the mediastinum, both as absolute and as relative values.

### Cytopathologic staging

EBUS-NA was performed in an outpatient setting using a flexible bronchoscope with a linear scanning transducer (BFUC180F, Olympus Optical Co Ltd., Tokyo, Japan). The bronchoscope has a convex distal probe capable of producing linear parallel scans of the mediastinal and peribronchial tissues and a working channel suitable for the performance of NA under direct ultrasound guidance. Local anesthesia and sedation were achieved using topical lidocaine spray and intravenous midazolam, propofol and/or fentanyl in accordance with standard recommendations [22]. Regional lymph node stations of the mediastinum and hilar regions (stations 2, 4, 7, 10 and 11) were systematically imaged and measured, and all nodes with a short-axis diameter of 5 mm or more were sampled with a 22-gauge needle (NA-201SX-4022, Olympus Optical Co Ltd.).

Samples were categorized according to whether they had been extracted from a normal node, showing lymphocytes and no neoplastic cells, or from a metastatic node, with groups of neoplastic cells. Aspirates containing only bronchial or blood cells, non-diagnostic atypical cells or insufficient material were considered inadequate. Every identified node was sampled at least three times, unless rapid on-site evaluation performed by a pathologist during the procedure confirmed the adequacy of the sample after the first or the second aspiration [23, 24].

Patients with negative EBUS-NA results were staged through EUS-NA, in left-sided tumors with abnormal lymph nodes by CT and/or FDG-PET in mediastinal regions 7, 8 or 9 [25–28]. Samples obtained through this procedure were categorized with the same criteria used for EBUS-NA. Patients with negative results in all the endoscopic procedures underwent cervical mediastinoscopy in accordance with guidelines [19, 20]. Systematic nodal dissection was performed during therapeutic surgery as the gold standard procedure for mediastinal staging.

### Statistical analysis

Statistical analyses were performed using the SPSS statistical software package version 18 (SPSS Inc., Chicago, IL, USA). Results obtained from categorical variables are expressed as absolute and relative frequencies, and results for continuous variables as means and standard deviations (SD) when the distribution was normal, or as medians and interquartile ranges (IQRs) when the distribution was not normal.

First, absolute SUVmax values for the primary tumor and the mediastinal node with highest FDG-PET uptake were measured, and the variability between hospitals assessed using one way ANOVA. Relative values of SUVmax in mediastinum (using SUVmax of the primary tumor as the reference) were calculated, both as a ratio (SUVmax mediastinum/SUVmax tumor) and as a subtraction (SUVmax mediastinum - SUVmax tumor), and the intercenter variability of these values was measured (one way ANOVA).

Second, the predictive power of absolute and relative SUVmax values for mediastinal malignancy was assessed, using pathology results as gold standard and applying logistic regression. The accuracies of different measures of SUVmax in the mediastinum were examined using ROC analyses and area under the curve (AUC). SUVmax cutoffs with maximal accuracy for the identification of mediastinal malignancy were defined, and their predictive powers for the identification of malignancy were calculated through univariate and multivariate logistic regression analyses. The results obtained in these analyses were expressed as crude and adjusted odds ratios (OR), with 95% confidence intervals (CI). All variables showing an association with the outcome variable (*p* < 0.10) in the univariate analysis were included in the multivariate models.

Finally, to determine the need for additional staging techniques after negative results in EBUS-NA, the sensitivity and NPV of this endoscopic technique for the diagnosis of mediastinal malignancy were assessed in patients at high and low risk according to their SUVmax values. Results were compared using the chi-square test. All tests used in the analyses were two-sided, and a *p* value of 0.05 or less was reported as statistically significant.

## Results

One hundred and twenty-one patients from five hospitals with a diagnosis of NSCLC were enrolled in the study over two years. Their median age was 65.7 years; adenocarcinoma was the most frequent tumor (52.9%), and a FDG-PET showed one or more hypermetabolic lymph nodes in the mediastinum in 77.7% of patients. The mean SUVmax of the primary tumor was 12.3 (SD 6.3) and the median SUVmax of the mediastinal node with the highest FDG-PET uptake was 3.9 (IQR 2.4-7.0). Mediastinal malignancy was diagnosed in 65 patients (53.7%), with EBUS-NA being the procedure which attained this diagnosis most frequently. Mediastinal lymph node involvement was confirmed through systematic nodal dissection performed during therapeutic surgery in six patients with negative endoscopic procedures and cervical mediastinoscopies (Table 1).

**Table 1:** Patient characteristics (*n* = 121)

Sociodemographic data	
Age (years), mean (SD)	65.7 (9.2)
Gender (female), *n* (%)	17 (14.0)
Tumor characteristics	
Pathology, *n* (%)	
Squamous-cell carcinoma	46 (38.0)
Adenocarcinoma	64 (52.9)
Large cell carcinoma	4 (3.3)
Non-specified non-small cell lung cancer	7 (5.8)
Tumor size, *n* (%):	
≤ 3 cm	46 (38.0)
> 3 cm and ≤7 cm	57 (47.1)
> 7 cm	18 (14.9)
F-18 fluorodeoxyglucose Positron emission tomography (FDG-PET)	
SUVmax tumor, mean (SD)	12.3 (6.3)
Single hypermetabolic image in the mediastinum, *n* (%)	50 (41.3)
Multiple hypermetabolic images in the mediastinum, *n* (%)	44 (36.4)
SUVmax mediastinum, median (IQR)	3.9 (2.4-7.0)
Staging of the mediastinum	
Malignancy extended to mediastinal lymph nodes	65 (53.8)
Technique diagnosing mediastinal malignancy	
EBUS-NA	53 (43.8)
EUS-NA or mediastinoscopy	6 (5.0)
Surgical lymphadenectomy	6 (5.0)

**Fig. 1:**
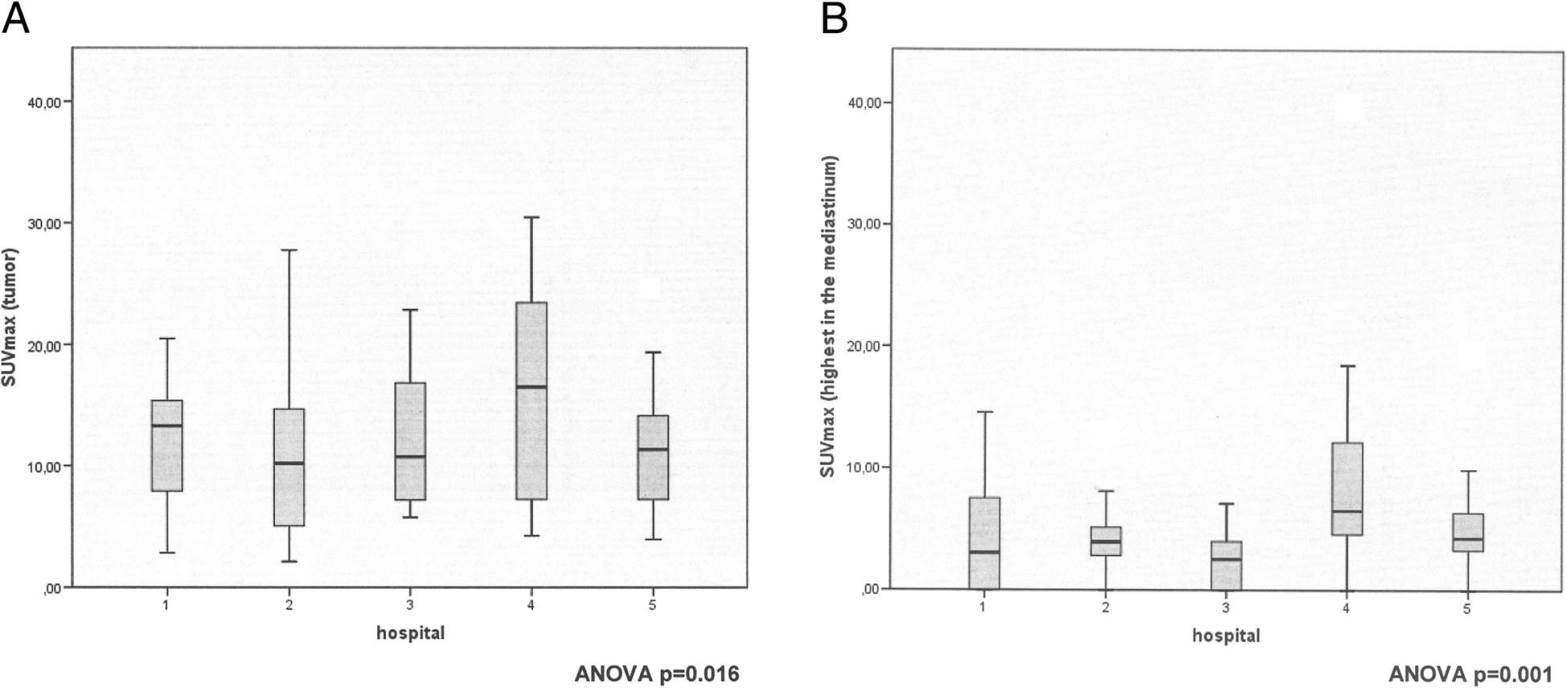
**a** Variability of FDG-PET between hospitals (absolute values): Maximum Standard Uptake Value (SUVmax) of the tumor (*p* =0.016, one way ANOVA). **b** Variability of FDG-PET between hospitals (absolute values): Highest maximum Standard Uptake Value (SUVmax) in the mediastinum (*p* < 0.001, one way ANOVA)

**Fig. 2:**
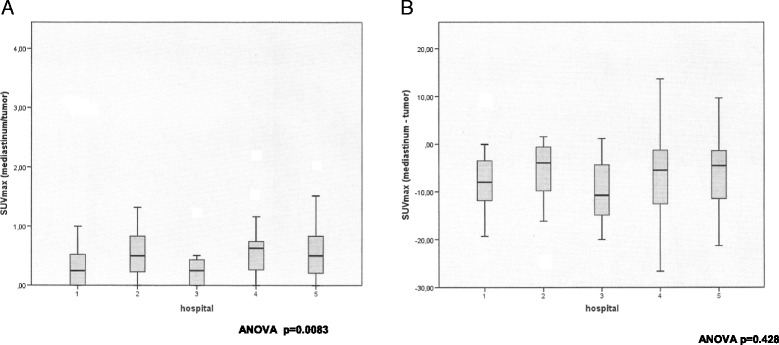
**a** Variability of FDG-PET between hospitals (relative values): Ratio highest maximum Standard Uptake Value (SUVmax) in the mediastinum/SUVmax tumor (*p* = 0.083; one way ANOVA). **b** Variability of FDG-PET between hospitals (relative values): Highest maximum Standard Uptake Value (SUVmax) in the mediastinum minus SUVmax tumor (*p* = 0.428; one way ANOVA)

Between-center FDG-PET variability in absolute SUVmax obtained from the tumor and the hypermetabolic image with the highest FDG uptake in the mediastinum was statistically significant (*p* = 0.016 and *p* < 0.001 respectively; one way ANOVA) (Figs. 1a and 1b). This variability in the mediastinum values disappeared when the SUVmax of the image with highest FDG uptake was adjusted for the SUVmax of the tumor, either as a ratio (SUVmax mediastinum/tumor) or as a subtraction (SUVmax mediastinum – tumor) (*p* = 0.083 and *p* = 0.428 respectively; one way ANOVA) (Figs. 2a and 2b).

**Fig. 3:**
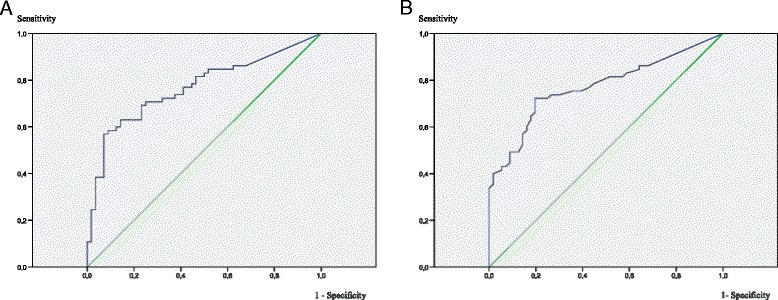
**a** Prediction of metastasis in the mediastinum in lung cancer by FDG-PET: Area under the curve (AUC) at ROC analysis for the maximum Standard Uptake Value (SUVmax) in mediastinum. Area under de curve (AUC 0.77 CI 0.69-0.86, *p* < 0.001). **b** Prediction of metastasis in the mediastinum in lung cancer by FDG-PET: Area under the curve (AUC) at ROC analysis for the maximum Standard Uptake Value (SUVmax) in mediastinum divided by the SUVmax of tumor (AUC 0.77 CI 0.68-0.85, *p* < 0.001)

SUVmax of the primary tumor did not show predictive power for identifying mediastinal extension of the disease (OR 0.98, 95%CI 0.92-1.04), but SUVmax in the mediastinum was clearly associated with malignancy when attaining values above the median (3.9). Equivalent results were found for the median values of the ratio between SUVmax in the mediastinum and SUVmax of the tumor (0.4) and the subtraction of this latter value from the SUVmax in the mediastinum (-6.1) (Table 2). Areas under the curve at ROC analysis for SUVmax in the mediastinum (AUC 0.77 CI 0.69-0.86, *p* < 0.001) (Fig. 3a) and for the ratio between this value and the SUVmax of the tumor (AUC 0.77 CI 0.68-0.85, *p* < 0.001) (Fig. 3b) were equivalent, confirming that the two measures were equally useful. The result of subtracting the SUVmax of the tumor from the highest SUVmax in the mediastinum showed a lower figure, still statistically significant (AUC 0.74 CI 0.6-0.83, *p* < 0.001). Cutoffs of 4 for SUVmax in the mediastinum and of 0.4 for the ratio between the SUVmax in the mediastinum and the tumor showed similar sensitivities, specificities and accuracies over 70% for the identification of mediastinal malignancy (Table 3). Accordingly, these two measures of SUVmax in the mediastinum may be considered equivalent, with the advantage of a greater homogeneity between centers when using the SUVmax mediastinum/tumor ratio.

**Table 2:** SUVmax values as a predictor of mediastinal malignancy

		Quartile categories	OR (95% IC)	*p*
SUVmax tumor	11.4 (SD 5.3)		0.98 (0.92-1.04)	0.469
SUVmax mediastinum	3.9 (IQR 2.4 - 7.0)	1st Q (reference) 2nd Q 3rd Q 4th Q	1 0.67 (0.22-2.01) 4.44 ( 1.49-13.26) 18.00 (4.38-74.01)	0.471 0.007 <0.001
$\frac{\mathrm{SUVmax}\kern0.5em \mathrm{Mediastinum}}{\mathrm{SUVmax}\kern0.62em \mathrm{Tumor}}$	0.4 (IQR 0.1 - 0.7)	1st Q (reference) 2nd Q 3rd Q 4th Q	1 1.06 (0.36-3.14) 3.89 (1.35-11.21) 18.52 (4.42-77.60)	0.919 0.012 <0.001
SUVmax Mediastinum *minus* SUVmax Tumor	[-6.1] (IQR [-11.7] – [-2.0])	1st Q (reference) 2nd Q 3rd Q 4th Q	1 2.58 (0.88-7.54) 3.57 (1.17-10.04) 23.15 (6.00-95.71)	0.083 0.016 <0,001

**Table 3:** Accuracy of cut-off SUVmax values for mediastinal malignancy, expressed as absolute value or as a ratio

	Sensitivity	Specificity	PPV	NPV	Accuracy
SUVmax mediastinum >4	72.3	80.4	81.0	71.4	76.0
$\frac{\mathrm{SUVmax}\kern0.5em \mathrm{Mediastinum}}{\mathrm{SUVmax}\kern0.62em \mathrm{Tumor}}>0.4$	70.8	73.2	75.4	68.3	71.9

Younger age and adenocarcinoma type were predictors of mediastinal involvement in NSCLC. The observation of a single hypermetabolic image in the mediastinum during FDG-PET did not reach statistical significance as a predictor of mediastinal malignancy (OR 2.34, 95% CI 0.89-6.22), but multiple FDG-PET hypermetabolic lymph nodes were significantly associated with the spread of NSCLC to the mediastinum (OR 3.87, 95% CI 1.40-10.66). SUVmax in the mediastinum above 4 and SUVmax mediastinum/tumor ratio above 0.4 showed similar predictive power for mediastinal malignancy (OR 10.68, 95% CI 4.55-25.10 and OR 6.62, 95% CI 2.98-14.69 respectively) (Table 4), which did not change significantly after adjusting for age, uni- and multilevel positive FDG-PET in the mediastinum and type of tumor in the multivariate analysis (OR 10.81, 95% CI 4.32-27.07 and OR 5.75, 95% CI 2.49-13.29 respectively).

**Table 4:** Univariate analysis of predictors for mediastinal malignancy

	OR (CI)	*p*
Age	0.95 (0.92-0.99)	.029
Gender (women)	2.31 (0.76-7.02)	.140
Tumor size	0.81 (0.48-1.37)	.435
Adenocarcinoma	2.45 (1.18-5.10)	.017
Single hypermetabolic node	2.34 (0.89-6.22)	.086
Multiple hypermetabolic nodes	3.87 (1.40-10.66)	.009
SUVmax Mediastinum > 4	10.68 (4.55-25.10)	<.001
$\frac{\mathrm{SUVmax}\kern0.5em \mathrm{Mediastinum}}{\mathrm{SUVmax}\kern0.62em \mathrm{Tumor}}>0.4$	6.62 (2.98-14.69)	<.001

EBUS-NA confirms an extension of NSCLC to the mediastinum in more that 15% of the cases independently of FDG-PET results in our study. Sensitivities and negative predictive values of EBUS-NA for the diagnosis of mediastinal malignancy, however, did not attain values above 90% for most of the subgroups categorized according to SUVmax in the mediastinum, with equivalent figures for SUVmax in the mediastinum and SUVmax mediastinum/tumor ratios (*p* > 0.05, chi square test) (Table 5). This finding supports the use of additional staging techniques such as EUS-NA and/or cervical mediastinoscopy to achieve accurate mediastinal staging previous to therapeutic surgery. In tumors with a SUVmax below the cutoff and diameters of 3 cm or less, however, the use of additional techniques in clinical staging in our study did not increase the presurgical sensitivity and negative predictive value, with equivalent results when there is none or one single hypermetabolic lymph node in the mediastinum, or multiple FDG-PET hot spots (*p* > 0.05, chi square test). These results suggest that in this clinical situation it may be possible to proceed to therapeutic surgery and systematic nodal dissection after a negative EBUS-NA exam.

**Table 5:** Ebus sensitivity and negative predictive value according to FDG-PET results

	SUV Cut-off	Tumor size	*n*	Mediastinal malignancyat EBUS	EBUS-NA Sensitivity	EBUS-NA NPV	Mediastinal malignancy at EUS-NA or mediastinoscopy	Mediastinal malignancy at systematic nodal dissection
SUVmax mediastinum	≤4	≤3 cm	22	4 (18.2%)	57.1%	83.3%	0 (0%)	3 (13.6%)
	≤4	>3 cm	41	8 (19.5%)	61.5%	84.8%	1 (2,4%)	4 (9.7%)
	>4	all	58	41 (70.7%)	87.2%	64.7%	5 (8.6%)	1 (1.7%)
$\frac{\mathrm{SUVmax}\kern0.5em \mathrm{mediastinum}}{\mathrm{SUVmax}\kern0.62em \mathrm{tumor}}$	≤0.4	≤3 cm	15	4 (26.7%)	66.7%	81.8%	0 (0%)	2 (13.3%)
	≤0.4	>3 cm	45	10 (22.2%)	76.9%	91.4%	1 (2.2%)	2 (2.2%)
	>0.4	all	61	39 (63.9%)	84.8%	68.2%	5 (8.2%)	2 (3.3%)

## Discussion

A highest SUVmax value in the mediastinum of over 4 proved to be a good predictor of mediastinal dissemination in patients with LC in our study. However, this FDG-PET measure showed variability between centers, a finding that limits its use a marker in staging for a specific hospital. This variability was corrected when the highest SUVmax value in the mediastinum was adjusted by the SUVmax of the primary tumor, either as a ratio (SUVmax mediastinum/tumor) or as a subtraction (SUVmax tumor - mediastinum). The ratio between the SUVmax in the mediastinum and the primary tumor emerged as the best relative predictor of nodal involvement by LC when attaining values over 0.4, showing a similar accuracy to SUVmax in the mediastinum expressed as an absolute value, without its intercenter variability.

The presence of significant intercenter differences in SUVmax values obtained from FDG-PET in LC patients is confirmed in our study, that shows that these differences can be corrected if the SUVmax of the primary tumor is used to adjust mediastinal values. Intercenter variability in SUVmax in the tumor and mediastinum has been reported in previous studies, depending on the scanner, reconstruction protocols [11, 12], and time between dose injection and imaging [14]. The use of the ratio between the SUVmax in the mediastinum and tumor (SUVmax mediastinum/tumor) was first proposed by Cerfolio and cols. as a way of correcting intercenter variability [13], and this relative value has been used successfully by other authors to identify mediastinal malignancy [14, 15]. Our study confirms the utility of the adjustment of SUVmax in the mediastinum using the SUVmax of the tumor, either as a ratio or as a subtraction, in order to avoid intercenter variability, allowing the comparison of results between centers.

The SUVmax mediastinum/tumor ratio emerged as the best predictor of mediastinal involvement in LC. This measure was not affected by intercenter variability, and attained a high AUC value, equivalent to the figure obtained by SUVmax when expressed as an absolute value and higher than the subtraction SUVmax mediastinum - tumor. The SUVmax mediastinum/tumor ratio showed an OR 6.62 (2.98-14.69) for mediastinal malignancy when above 0.4, similar to the OR of 10.68 (4.55-25.10) found for absolute values of SUVmax in the mediastinum when above 4. This SUVmax mediastinum/tumor ratio cutoff attained a sensitivity of 70.8 and an accuracy of 71.9% for the identification of malignancy, similar to the results achieved using absolute values. The cutoffs proposed in previous studies for SUVmax mediastinum/tumor ratios for attaining a sensitivity above 70% have ranged between 0.28 and 0.56 [13–15], and our results confirm that the use of a 0.4 cutoff, inside this range, attains a sensitivity above this figure. To attain an equivalent sensitivity for the identification of malignancy, absolute SUVmax values in the mediastinum ranging between 2.5 and 5.3 have been proposed [8–10, 17], but they remain unsuitable for intercenter comparisons.

Adenocarcinoma type, younger age and multiple FDG-PET hypermetabolic lymph nodes, were significantly associated with extension to the mediastinum of NSCLC in the present study, but the predictive power of the SUVmax mediastinum/tumor ratio for malignant extension did not change after adjustment for these variables. However, in spite of its usefulness for the identification of mediastinal extension in LC, FDG-PET did not obtain an accuracy above 80%, and sampling of the lymph nodes in the mediastinum is required for the confirmation of positive imaging results and the identification of malignancy in patients who have negative FDG-PET exams but are at a high risk of regional extension [4, 5, 7, 17]. EBUS-NA is the first approach for mediastinal sampling after FDG-PET, and in the present study it increased the accuracy of clinical staging independently of the FDG-PET results. EBUS-NA only attained a sensitivity over 80% when FDG-PET value was above the cutoff, however, and showed a NPV below 70% in this subgroup of patients. These results are not high enough to obviate the need for additional staging procedures before surgery such as EUS-NA and mediastinoscopy in order to minimize understaging, except in patients with tumors with a diameter of 3 cm or below, where the NPV of clinical staging with EBUS-NA did not increase with additional sampling through EUS and/or mediastinoscopy. These findings support the recommendation in current guidelines of mediastinal sampling using EBUS-NA after FDG-PET in NSCLC [19, 20], and suggest that this endoscopic technique may be enough for presurgical staging in patients with lung tumors up to 3 cm in diameter, when the highest SUVmax mediastinum/tumor ratio was below 0.4.

## Conclusion

SUVmax mediastinum and the SUVmax mediastinum/tumor ratio are both good predictors of mediastinal malignancy in NSCLC, and both show accuracies over 70% at values above 4 and 0.4 respectively. When SUVmax is measured as a ratio it is not influenced by intercenter variability, and allows comparisons between hospitals without additional adjustments. The presence of multiple FDG-PET hypermetabolic lymph nodes in the mediastinum predicts the regional extension of the tumor, but presents a much lower OR than SUVmax, and thus has a lower diagnostic power than the measurement of SUV. However, most FDG-PET results obtained in the mediastinum during LC staging would need to be confirmed through semi-invasive techniques, given that the predictive values of the technique remain below 90%. EBUS-NA increases the accuracy of FDG-PET, and may be enough for presurgical staging in small tumors with low SUVmax mediastinum/tumor values.

## Abbreviations

AUC: Area under the curve
CT: computed tomography
EBUS-NA: Endobronchial ultrasonography with needle aspiration
EUS-NA: esophageal ultrasound with needle aspiration
FDG-PET: F-18 fluorodeoxyglucose positron emission tomography
IQR: interquartile range
NPV: negative predictive value
ROC: Receiver Operating Characteristic
SUVmax: maximum Standard Uptake Value
